# Treatment patterns and outcomes in patients with multiple myeloma in second relapse in Colombia (freedomm): a multicenter observational study

**DOI:** 10.3389/fonc.2026.1831845

**Published:** 2026-07-01

**Authors:** Guillermo Quintero, Sandra Aruachan, Jair Figueroa Emiliani, Kenny Galvez, Luis Antonio Salazar, Constanza Otero Venegas, Diana Buitrago, Humberto Martínez-Cordero, Juliana Saavedra, Maria Fernanda Vargas

**Affiliations:** 1 Fundación Santa Fe de Bogotá, Bogotá, Colombia; 2 Instituto Médico de Alta Tecnología Oncomédica, Montería, Colombia; 3 Hospital Mayor Méderi, Bogotá, Colombia; 4 Hospital Pablo Tobón Uribe, Medellín, Colombia; 5 Hematology and Hematopoietic Stem Cell Transplantation Unit, Clinica FOSCAL, Floridablanca, Santander, Colombia; 6 Facultad de Ciencias de la Salud, Universidad Autónoma de Bucaramanga, Bucaramanga, Colombia; 7 Real-World Evidence – Epidemiology & Clinical Data Management, Latin America, Central America and the Caribbean, Bogotá, Colombia; 8 Centro de Excelencia en Mieloma Múltiple - Unidad de Hemato-Oncología, Instituto Nacional de Cancerología, Bogotá, Colombia; 9 Unidad de Hematología y Trasplante de Médula Ósea, Hospital Militar Central, Bogotá, Colombia; 10 Sanofi, Bogotá, Colombia

**Keywords:** multiple myeloma, treatment patterns, real-world evidence, effectiveness, safety

## Abstract

**Introduction:**

Treatment selection for patients with relapsed Multiple Myeloma (MM) is highly individualized and characterized by diverse treatment patterns. While substantial evidence exists regarding relapsed MM in high-income settings, data on treatment patterns and clinical outcomes at second relapse in Latin American populations remain limited. This study describes the clinical characteristics, treatment patterns, effectiveness, and safety of third-line therapy in patients with MM experiencing a second relapse in routine clinical practice across five Colombian institutions.

**Methods:**

This multicenter retrospective observational study included medical records from five specialized institutions in Colombia. Patients with MM who experienced a second relapse and initiated third-line therapy between 2013 and 2022 were included. Baseline characteristics, treatment patterns, and the effectiveness and safety of third-line therapy were analyzed descriptively.

**Results:**

The study included 84 participants with multiple myeloma who experienced a documented second relapse. The median age at diagnosis was 63.5 years (IQR 57.0 - 70.0), 56% were male, and bone disease and anaemia were the most frequent presenting features. The median time from diagnosis to second relapse was 3.2 years (IQR 1.8 - 4.9). First-line therapy was mainly bortezomib-based, most frequently the combination of bortezomib, cyclophosphamide, and dexamethasone (36.9%). Second-line treatment shifted toward lenalidomide-based regimens, with lenalidomide plus dexamethasone administered to 29.8% of patients. Autologous stem cell transplantation was performed in 36.9% of patients. Third-line treatment patterns were diverse, with greater use of novel agent–based combinations, including regimens containing daratumumab and carfilzomib. In the third-line setting, the overall response rate was 47.1%. The median overall survival from initiation of treatment at second relapse was 27.5 months, while the median progression-free survival and time to treatment failure were 14.6 months and 5.94 months, respectively. Treatment-emergent adverse events (TEAEs) occurred in 11.9% of patients, mostly moderate.

**Conclusion:**

In this real-world study of patients with multiple myeloma treated in the third-line setting after second relapse, treatment patterns were heterogeneous, with increasing use of novel agent–based combinations. An objective response was achieved in nearly half of the patients, with a median overall survival of 27.5 months and a median progression-free survival of 14.6 months. Treatment-emergent adverse events were infrequently reported and predominantly hematologic. No health-related quality of life data were available, highlighting an important gap in routine clinical practice.

## Introduction

Multiple myeloma (MM) is a malignant disorder of plasma cells that leads to excessive monoclonal immunoglobulin production and progressive organ damage. As of 2022, MM accounted for nearly 1.0% of all new cancer diagnoses and 1.1% of cancer-related deaths globally (1). The global incidence of MM has continued increasing, particularly in high-income countries, influenced by population aging and improved diagnostic practices (2, 3). In Latin America (LATAM), the burden of MM has also increased, although disparities in access to care and treatment remain a major concern (4). Survival outcomes in the region remain lower compared to high-income countries, reflecting persistent inequities in access to timely diagnosis and effective therapies (4).

While novel agents, including proteasome inhibitors (PI), immunomodulators, and other new therapeutic strategies, have improved survival in developed settings, such progress has not been equitably reflected in LATAM, where access to those therapies is limited (4, 5). Most patients with MM respond to first-line therapy, but the disease remains incurable, and relapses, defined as *“disease progression accompanied by new or worsening damage”* (6), are inevitable. With each recurrence, the duration of response tends to shorten, and treatment options become more limited (7, 8).

Therapy selection in relapsed MM is guided by several factors: prior treatment history and response, patient age, previous toxicities, performance status, comorbidities, and cytogenetic profile (5). Despite a growing body of clinical trial and Real-World Evidence (RWE) on relapsed MM, most available data derive from studies conducted in North America and Europe, where Latin American and Hispanic populations remain underrepresented (9). Consequently, evidence describing treatment patterns and outcomes in patients with MM at second relapse remains scarce in LATAM (10), where access to newer therapies is limited and treatment sequencing differs from high-income settings (11).

In LATAM, the management of patients with MM at second or later relapse is strongly influenced by healthcare system constraints, delayed regulatory approval, and limited reimbursement of novel anti-myeloma agents (10). In Colombia, particularly, variations in healthcare coverage, access to novel anti-myeloma agents, and institutional treatment practices may further influence therapeutic decision-making and clinical outcomes in later treatment lines. RWE from regional observational studies and targeted literature reviews indicated that treatment at advanced relapse stages frequently relies on proteasome inhibitor and immunomodulatory drug-based regimens, most commonly bortezomib- and lenalidomide-based combinations, with more limited and heterogeneous access to monoclonal antibodies such as daratumumab (10, 12, 13). In this context, reuse of agents administered in earlier lines of therapy is common, largely because access to newer treatments remains inconsistent across countries and healthcare sectors (10, 12). Marked differences have been reported between public and private institutions, with patients treated in private settings more likely to receive triplet regimens and novel agents, associated with improved clinical outcomes while comparing with those managed in public healthcare systems (10, 14). Understanding clinical characteristics, treatment patterns, and outcomes in this setting is crucial to support context-specific clinical and policy decisions.

This study aimed to describe the characteristics of patients, treatment patterns, effectiveness, and safety in patients with MM experiencing their second relapse, as observed in routine clinical practice in five healthcare centers in Colombia.

## Materials and methods

**Figure 1:**
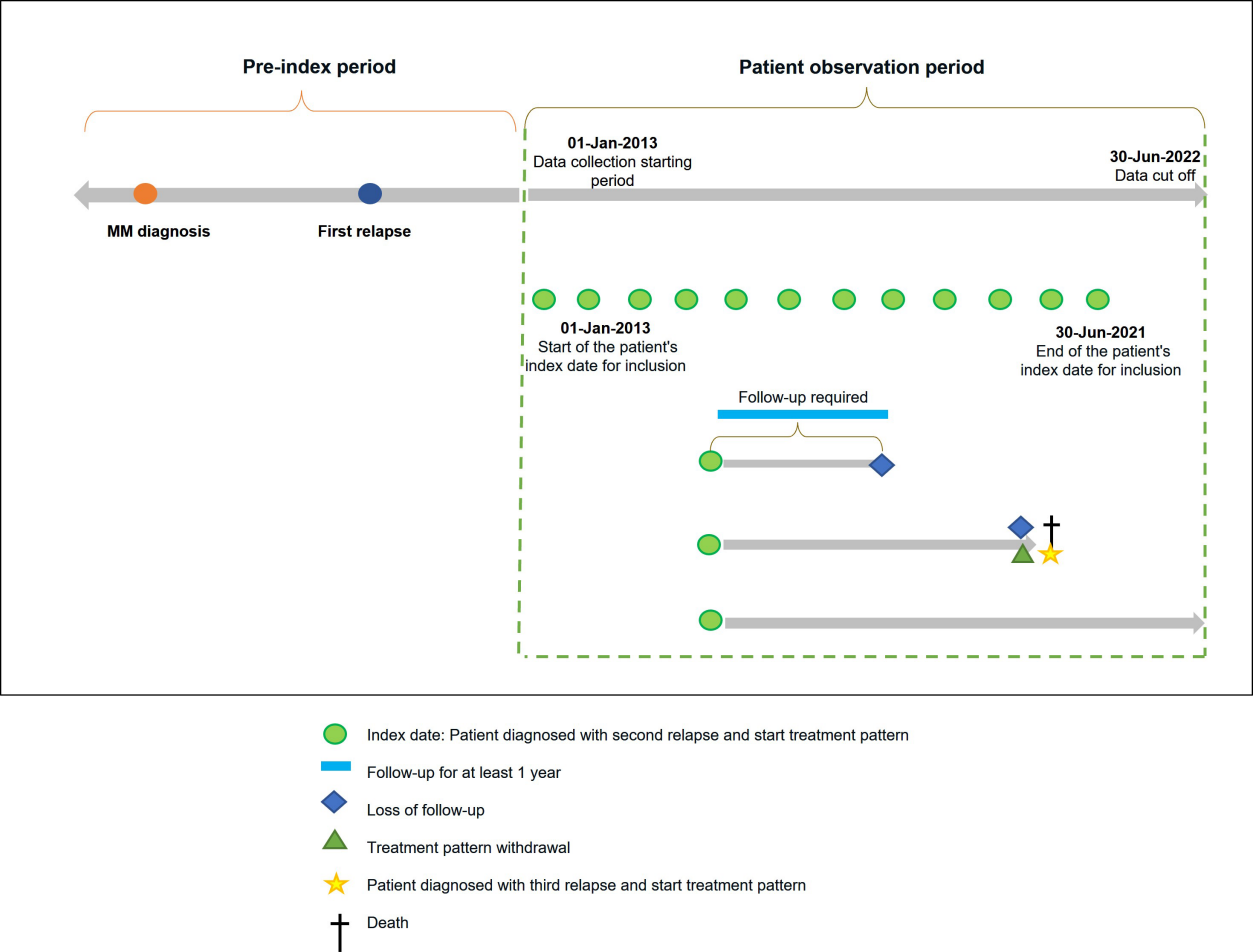
Study design.

We conducted a multicenter, retrospective observational study in five oncology-specialized institutions in Colombia, using routinely collected clinical data from eligible patients between January 1, 2013, and June 30, 2022 (**Figure 1**).

The study included adult patients (≥18 years) diagnosed with MM between January 1, 2013, and June 30, 2021. Patients were eligible if they had received at least two prior treatment lines (including both lenalidomide and a PI), experienced a second relapse, initiated a third-line therapy, and had at least one year of follow-up after treatment initiation.

Exclusion criteria included diagnosis of other plasma cell or immunoglobulin-related disorders, receipt of only palliative care or no treatment after second relapse, or participation in interventional clinical trials for MM treatment. The final sample size corresponded to the total number of eligible patients identified across all participating institutions.

Medical records were reviewed to collect data from three defined periods: the pre-index period (from MM diagnosis to the index date), the index date (defined as the day when the patient started the treatment pattern for the second relapse), and the follow-up period (from the index date to June 30, 2022, or treatment discontinuation, third relapse, death, or loss to follow-up).

We collected data on patients’ demographic (gender, age, region of residency, healthcare affiliation), clinical characteristics (MM staging), and treatment history (autologous stem cell transplantation (ASCT), and first-line therapy from the pre-index period), as well as information on the first relapse and second-line therapy from this period. Additionally, we gathered information related to the second relapse, treatment patterns, and adverse events at the index date. Clinical relapse was defined accordingly with the International Myeloma Working Group (15). Biochemical relapse was defined as patients who present an increase of serum and/or urine monoclonal protein alone, with no other clinical symptoms (16).

Time-to-event endpoints were defined as follows. Progression-free survival (PFS) was defined as the time from initiation of treatment for second relapse to documented disease progression or death from any cause, whichever occurred first; patients without an event were censored at the last adequate disease assessment or data cutoff.

Overall survival (OS) was defined as the time from treatment initiation for second relapse to death from any cause, with patients censored at the last known date alive or data cutoff. Time to treatment failure (TTF) was defined as the time from third-line treatment initiation to treatment discontinuation for any reason, including progression, toxicity, death, or other causes.

Given the heavily pre-treated population, death without prior progression or treatment change may represent a competing event; however, competing risks were not explicitly modeled, and Kaplan–Meier methods were used for all analyses.

ORR was calculated in the response-evaluable population, defined as patients with at least one post-baseline response assessment prior to subsequent therapy. Patients who progressed, died, or discontinued treatment before response assessment were considered non-evaluable.

Safety was evaluated by the frequency of treatment-emergent adverse events (TEAEs), serious adverse events (TESAEs), and treatment discontinuation due to TEAEs. Definitions of all outcomes are provided in **Supplementary File 1**.

As part of the exploratory analyses, we explored outcomes based on the time from diagnosis to second relapse (<6, 6-12, 12–24 and > 24 months). These subgroup comparisons aimed to identify potential differences in response rates and survival outcomes across clinically relevant strata.

To minimize bias, only institutions with rigorous electronic medical record systems and documentation standards were included. Consecutive sampling was used to reduce selection bias. To validate responses between sites, a random sample of medical records were compared against data registered in the platform during monitoring visits. No imputation was applied for missing data; missingness was reported per variable.

Descriptive statistics were used for all variables. Categorical variables were summarized using frequencies and percentages, which were calculated over the number of patients with available data (non-missing). Continuous variables were described using medians and interquartile ranges (IQR), as well as 95% confidence intervals (95% CI), when applicable.

The study protocol was reviewed and approved by the institutional review boards or ethics committees at all participating centers. The study adhered to the Declaration of Helsinki, Good Clinical Practice guidelines, and local regulations. This report was written in accordance with The Strengthening the Reporting of Observational Studies in Epidemiology (STROBE) statement.

## Results

### Demographic and clinical characteristics

Of the 98 patients initially identified, 14 were excluded due to unmet eligibility criteria, primarily because they had not experienced a clinical relapse after at least two prior treatment lines (including lenalidomide and a proteasome inhibitor) or did not have a documented second relapse within the predefined study period. As a result, 84 patients were included in the full analysis set.

The median age at diagnosis was 63.5 years (IQR 57.0 - 70.0), and 56.0% of patients were male. Most patients were residents of Bogotá D.C. (32.5%) and the department of Córdoba (21.7%), and the majority were affiliated with the contributory healthcare regime (72.6%) (**Table 1**).

**Table 1:** Demographic and clinical characteristics of participants at diagnosis and second relapse.

Characteristic	Value
Age at diagnosis (years)	
Mean (SD)	62.3 (11.7)
Median (IQR)	63.5 (57.0 - 70.0)
Min - Max	28.0 - 87.0
Age at second relapse (years)	
Mean (SD)	66.3 (11.5)
Median (IQR)	67.0 (60.9 - 73.6)
Min - Max	30.4 - 88.9
Time from diagnosis to second relapse (years)	
Mean (SD)	3.6 (2.1)
Median (IQR)	3.2 (1.8 - 4.9)
Min - Max	0.6 - 11.2
Sex, N (%)	
Female	37 (44.0%)
Male	47 (56.0%)
Affiliation regime, N (%)	
Contributory	61 (72.6%)
Subsidized	13 (15.5%)
Special	9 (10.7%)
No affiliation	0
Unknown	1 (1.2%)
Defining symptom of MM at diagnosis, N (%)	
Hypercalcemia	14 (16.7%)
Renal failure	30 (35.7%)
Anaemia	54 (64.3%)
Bone disease	57 (67.9%)
Other	5 (6.0%)
Unknown	1 (1.2%)
International Staging System (ISS) at the MM diagnosis, N (%)	
I	10 (11.9%)
II	23 (27.4%)
III	21 (25.0%)
Unknown	30 (35.7%)
International Staging System (ISS) at first-relapse, N (%)	
I	5 (6.0%)
II	22 (26.2%)
III	18 (21.4%)
Unknown	39 (46.4%)
Bone lesion at diagnosis, N (%)	
Yes	59 (70.2%)
No	10 (11.9%)
Unknown	15 (17.9%)
Bone lesion at first relapse, N (%)	
Yes	41 (48.8%)
No	23 (27.4%)
Unknown	20 (23.8%)
Patients with at least one comorbidity at diagnosis, N (%)	
Yes	37 (44.0%)
No	37 (44.0%)
Unknown	10 (11.9%)
Comorbidities at diagnosis description, N(%) ^*^	
Arterial hypertension	23 (62.2%)
Diabetes mellitus	6 (16.2%)
Chronic liver disease	2 (5.4%)
Chronic kidney disease	1 (2.7%)
Kidney replacement therapy^**^	1 (100.0%)
Solid neoplasm	1 (2.7%)
Other	29 (78.4%)
Patient with any medical history at first relapse, N (%)	
Yes	59 (70.2%)
No	25 (29.8%)
Unknown	0 (0.0%)
Patient with any medical history at second relapse, N (%)	
Yes	61 (72.6%)
No	22 (26.2%)
Unknown	1 (1.2%)
Patient with any medical history at third relapse, N (%)	
Yes	35 (41.7%)
No	14 (16.7%)
Unknown	35 (41.7%)

*One patient can have more than one comorbidity.

**Percentages are calculated using patients with chronic kidney disease as denominator.

Missing values are only reported for variables that had missing data.

The most frequent symptoms at diagnosis were bone disease (67.9%) and anemia (64.3%), with 70.2% of patients presenting with lytic bone lesions. Comorbidities were documented in 44.0% of patients, most commonly hypertension (62.2%). According to the International Staging System (ISS), 11.9% of patients were classified as stage I, 27.4% as stage II, and 25.0% as stage III, while ISS stage was unknown in 35.7% of cases. ECOG performance status at diagnosis was not consistently documented, with the majority of records classified as unknown. The median time from diagnosis to second relapse was 3.2 years (IQR 1.8–4.9), and the median age at second relapse was 67.0 years (IQR 60.9 - 73.6) (**Table 1**).

### Treatment patterns

The median time from diagnosis to the initiation of first-line therapy was 21.0 days (IQR 2.0 – 51.0). As shown in **Table 2**, first-line treatment primarily consisted of bortezomib-based combinations, reflecting standard induction practice. The most frequently used regimen was bortezomib plus cyclophosphamide and dexamethasone, administered to 36.9% of patients, followed by bortezomib, dexamethasone, and thalidomide (16.7%) and bortezomib, melphalan, and prednisone (10.7%). A heterogeneous group of less frequently prescribed combinations accounted for 23.8% of first-line regimens and was grouped under “other”.

**Table 2:** Treatment patterns and treatment combinations for first, second and third line of therapy.

Treatment characteristics	First-line therapy	Second-line therapy	Third-line therapy
Patient receiving any medication, N (%)			
Yes	84 (100.0%)	84 (100.0%)	84 (100.0%)
No	0 (0.0%)	0 (0.0%)	0 (0.0%)
Treatment regimen N (%)	N = 84	N = 84	N = 84
Bortezomib + Cyclophosphamide + Dexamethasone	31 (36.9%)	3 (3.6%)	1 (1.2%)
Bortezomib + Dexamethasone + Thalidomide	14 (16.7%)	1 (1.2%)	1 (1.2%)
Bortezomib + Melphalan + Prednisone	9 (10.7%)	1 (1.2%)	2 (2.4%)
Bortezomib + Dexamethasone	7 (8.3%)	2 (2.4%)	1 (1.2%)
Bortezomib + Dexamethasone + Lenalidomide	2 (2.4%)	10 (11.9%)	6 (7.1%)
Dexamethasone + Lenalidomide	1 (1.2%)	25 (29.8%)	2 (2.4%)
Bortezomib + Daratumumab + Dexamethasone	0 (0.0%)	1 (1.2%)	5 (6.0%)
Carfilzomib + Dexamethasone + Lenalidomide	0 (0.0%)	12 (14.3%)	8 (9.5%)
Cyclophosphamide + Dexamethasone + Lenalidomide	0 (0.0%)	7 (8.3%)	2 (2.4%)
Daratumumab + Dexamethasone + Lenalidomide	0 (0.0%)	1 (1.2%)	9 (10.7%)
Carfilzomib + Dexamethasone	0 (0.0%)	0 (0.0%)	9 (10.7%)
Other regimens	20 (23.8%)	21 (25.0%)	38 (45.2%)

1L, First-line therapy; 2L, Second-line therapy; 3L, Third-line therapy. *Percentages are calculated using the total number of prescriptions (not patients) as denominator, a single patient might receive more than one medication.

Certain treatments for multiple myeloma are not included in the table because they were not found in the analyzed cohort (such as Belantamab mafodotin-blmf, Cisplatin, Elotuzumab, Etoposide, Ixazomib, Melphalan flufenamide, Panobinostat, Selinexor).

Second-line therapy showed a shift toward lenalidomide-based regimens, with dexamethasone plus lenalidomide being the most frequently used combination (29.8%). Other commonly prescribed regimens included carfilzomib, dexamethasone, and lenalidomide (14.3%); bortezomib, dexamethasone, and lenalidomide (11.9%); and cyclophosphamide, dexamethasone, and lenalidomide (8.3%). Other regimens represented 25.0% of second-line treatments.

Autologous stem cell transplantation (ASCT) was performed in 36.9% of patients. Among those receiving maintenance therapy after ASCT, lenalidomide was the most commonly used agent (45.5%), followed by thalidomide (18.2%) and dexamethasone (15.2%) (**Supplementary Table 1**). Information on the number of induction cycles before ASCT and the depth of response at the time of transplantation was not consistently available and was therefore not analyzed.

In the third-line setting, treatment regimens became more heterogeneous, with increased use of novel agent–based combinations. The most frequently reported regimens were daratumumab, dexamethasone, and lenalidomide (10.7%), carfilzomib plus dexamethasone (10.7%), followed by carfilzomib, dexamethasone, and lenalidomide (9.5%) and bortezomib, dexamethasone, and lenalidomide (7.1%). The proportion of regimens categorized as “other” increased substantially in third-line therapy, accounting for 45.2%, reflecting wide variability and lower frequencies of individual combinations.

### Effectiveness of the treatment patterns

Effectiveness outcomes for all treatment regimens administered in the third-line setting are summarized in **Table 3**. The ORR, defined as the proportion of patients achieving sCR, CR, VGPR, or PR, was 47.1% (24 patients).

**Table 3:** Effectiveness of treatment patterns for second relapse.

Effectiveness	All treatments
Response to third-line therapy, N (%)	
ORR*	24 (47.1%)
OS with third-line therapy**	
Median (95% CI)	27.5 (19.8 - NE)
PFS with third-line therapy***	
Median (95% CI)	14.6 (9.4 - 22.3)
TTF with third-line therapy****	
Median (IQR)	5.94 (4.19 - 8.86)
Ongoing therapy	9

*ORR, percentage of patients with either sCR, CR, VGPR or PR of patients with MM treated with the different treatment patterns for second relapse. **OS, time from the date of start of second-relapse treatment to the date of death from any cause. ***PFS= time from the date of start of third-line therapy to the date of progressive disease or death from any cause. ****TTF, time from the date of start of third-line therapy until treatment discontinuation due to disease progression, treatment intolerance, or other reasons. NE, Not estimable, the upper confidence limit could not be calculated due to censoring.

**Figure 2:**
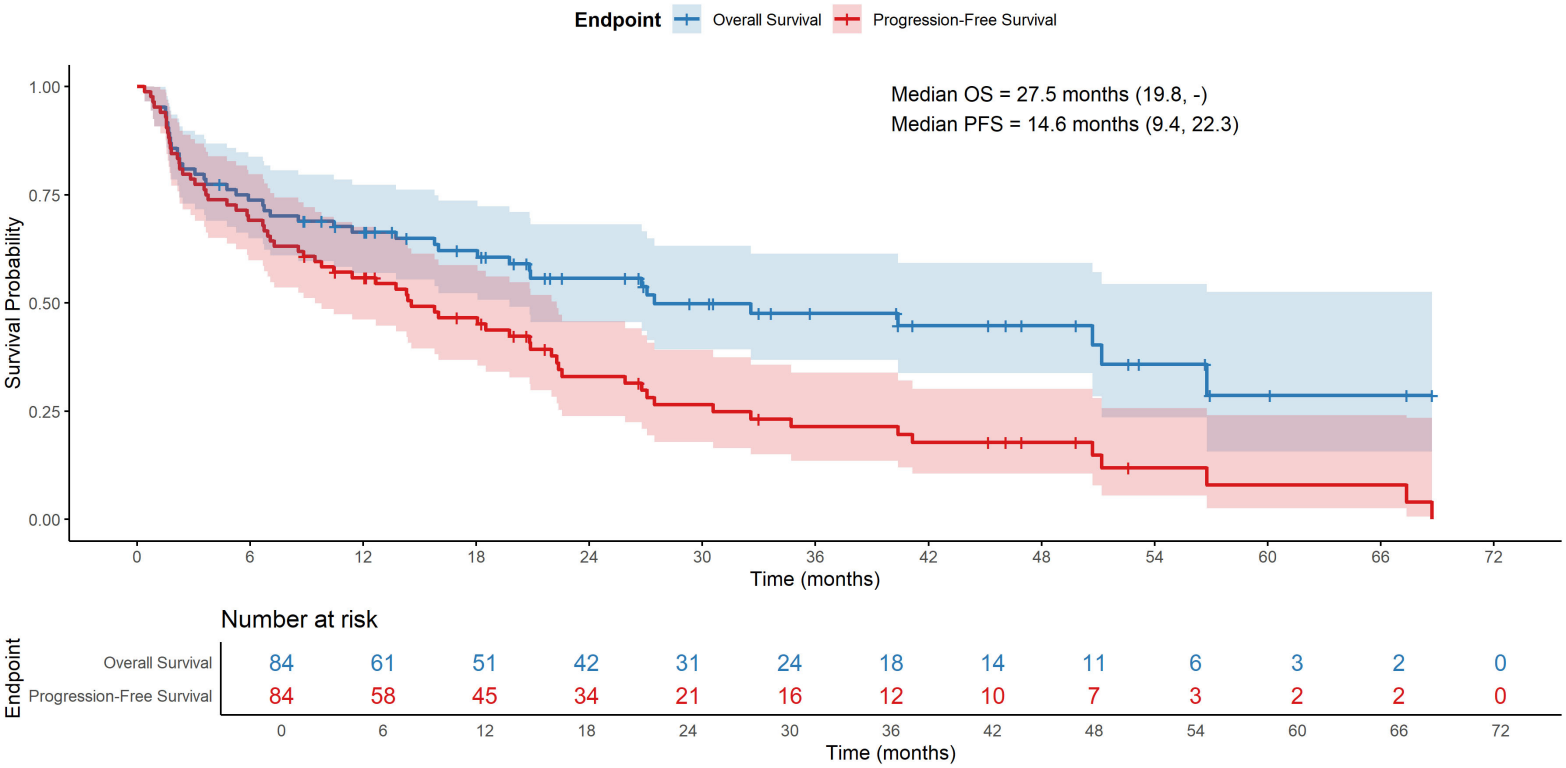
Overall Survival of patients with MM in second relapse in months.

With respect to survival outcomes, the median OS from the initiation of treatment for the second relapse was 27.5 months (95% CI: 19.8–NE) (**Figure 2**), indicating that the upper bound of the confidence interval could not be estimated because of censoring at the time of analysis.

The median PFS, measured from the start of third-line therapy to disease progression or death from any cause, was 14.6 months (95% CI: 9.4–22.3). The median TTF, defined as the interval from the start of third-line therapy to treatment discontinuation due to disease progression, treatment intolerance, or other reasons, was 5.94 months (95% CI: 4.19–8.86).

At the time of data cutoff, nine patients remained on treatment, indicating continued therapy beyond the observed follow-up period.

Results stratified by time to second relapse are presented in **Supplementary Table 2**.

### Safety of the treatment patterns and QoL

TEAEs were reported in 11.9% of patients receiving third-line therapy. The most frequently reported TEAEs were hematologic events, such as blood and lymphatic system disorders (54.5%), particularly anemia and neutropenia (both 18.2%) (**Table 4**). Most TEAEs were moderate (70.0%), while 30.0% were classified as serious.

**Table 4:** Treatment-emergent adverse events for treatment patterns for second relapse.

Parameters	N (%)
Patient with any adverse event, N (%)	
Yes	10 (11.9%)
No	74 (88.1%)
Adverse event description, N (%)*	
Blood and lymphatic system disorders	6 (54.5%)
Anaemia	2 (18.2%)
Febrile neutropenia	1 (9.1%)
Neutropenia	2 (18.2%)
Thrombocytopenia	1 (9.1%)
Cardiac disorders	1 (9.1%)
Atrial fibrillation	1 (9.1%)
Infections and infestations	1 (9.1%)
Urinary tract infection	1 (9.1%)
Nervous system disorders	1 (9.1%)
Cerebral venous sinus thrombosis	1 (9.1%)
Respiratory, thoracic, and mediastinal disorders	1 (9.1%)
Cough	1 (9.1%)
Vascular disorders	1 (9.1%)
Distributive shock	1 (9.1%)
TEAEs description, N (%)*	
Serious	3 (30.0%)
Resulted in death **	1 (33.3%)
Life-threatening **	1 (33.3%)
Required hospitalization **	3 (100.0%)
Caused persistent or significant ** disability/incapacity	1 (33.3%)
A birth defect or congenital anomaly was involved **	0 (0.0%)
TEAEs severity, N (%)*	
Mild	1 (10.0%)
Moderate	7 (70.0%)
Severe	2 (20.0%)
TEAEs outcome, N (%)*	
Recovered/Resolved	3 (30.0%)
Recovered/Resolved with sequelae	0 (0.0%)
In recovery/In resolution	5 (50.0%)
Not recovered/Not resolved	1 (10.0%)
Fatal	1 (10.0%)
Third-line therapy action related to the TEAE, N (%)*	
None	5 (50.0%)
Dose adjustment	2 (20.0%)
Treatment withdrawal	1 (10.0%)
Treatment interruption/discontinuation	2 (20.0%)

A patient can have one or more adverse event reported under a given system organ class. Percentages are calculated using the non-missing as the denominator. *Percentages are calculated using the number of total TEAEs as the denominator. A patient may have more than one TEAE**Percentages are calculated using the number of total serious TEAEs as the denominator. A patient may have more than one TEAE.

Among serious TEAEs, all required hospitalization, one resulted in death, and one resulted in significant disability. TEAEs led to dose adjustment (20.0%), treatment interruption (20.0%), or withdrawal (10.0%). A detailed summary of TEAEs is provided in **Table 4**.

Health-related quality of life was not assessed at diagnosis or at first repalpse in any patient. Consequently, no validated QoL instruments or scores were available for analysis.

## Discussion

This real-world study presents comprehensive insights into treatment patterns, effectiveness, and safety in patients with MM in second relapse, previously exposed to lenalidomide and a proteasome inhibitor, treated across five specialized healthcare institutions in Colombia. Third-line treatment regimen varied considerably, with lenalidomide, carfilzomib, bortezomib, and daratumumab among the most frequently prescribed agents, in addition to steroids. Across treatment types, the ORR was 47.1%, with a median OS of27.5 months, and a median PFS of 14.6 months. The median TTF was 5.94 months, reflecting early treatment discontinuation despite longer survival outcomes. More than half of the patients experienced disease progression or relapse on third-line therapy, with relapse and death being the most common reasons for treatment discontinuation. TEAEs were observed in approximately 12.0% of patients receiving third-line therapy, most of which were moderate in 70.0% of the cases and serious in 30.0% of these ones.

Patient demographics were consistent with global and regional reports. In our study, 56.0% of patients were male, with a mean age of 63.5 years at diagnosis, aligning with international data where MM predominantly affects older adults and with reported median ages at diagnosis in LATAM of approximately 60–65 years (2, 17). The average time from diagnosis to second relapse was 3.2 years, with a median age of 67 at relapse. Compared to reports from other regions, such as Spain where the median age at relapse is higher (69 years) (18) and the United Kingdom (U.K.) where the time from diagnosis to first relapse and from first to second relapse is shorter (19 months, 10 months respectively) (19), our cohort appears slightly younger with relatively prolonged disease control during early treatment phases.

Treatment patterns reflected variability in practice and evolving access to therapies. First-line regimens were predominantly bortezomib-based combinations, particularly cyclophosphamide and dexamethasone, consistent with standard induction approaches (20). In second-line treatment, there was a shift towards lenalidomide-based regimens, particularly lenalidomide plus dexamethasone. These findings contrast with earlier LATAM registries, such as HOLA study, where chemotherapy and the use of thalidomide-based regimens were more common and access to novel agents was limited (21). However, they are consistent with more recent evidence showing increasing use of proteasome inhibitors and immunomodulatory drugs across the region, although disparities in access persist (4, 10).

In third-line therapy, no single regimen exceeded 15% of prescriptions, underscoring the high degree of heterogeneity in treatment selection. Among the most frequently used regimens were daratumumab-lenalidomide-dexamethasone, carfilzomib-dexamethasone, and carfilzomib-lenalidomide-dexamethasone. Notably, a substantial proportion of patients (45.2%) received regimens categorized as “other”, limiting our ability to evaluate the impact of specific induction regimens on relapse timing and response to subsequent therapies. This heterogeneity also reflects the absence of standardized treatment pathways in this clinical setting, highlighting an important area for future research, particularly the development of context-specific recommendations to guide treatment decisions in real-world settings.

A shift in drug utilization was observed across treatment lines. Lenalidomide and bortezomib were widely used in earlier lines, while their relative use declined in third-line therapy, with increasing incorporation of newer agents such as carfilzomib and daratumumab. Additional therapies including pomalidomide, bendamustine, and liposomal doxorubicin also emerged in third-line settings. Similar patterns have been observed in the United States (U.S.), where novel agents (such as carfilzomib, pomalidomide, and daratumumab) are increasingly adopted in later treatment lines, while the use of bortezomib and lenalidomide, previously common in earlier lines, has declined as patients experience disease progression (22).

ASCT was performed in 36.9% of patients, a higher proportion than earlier LATAM reports (between 26.9% and 33.9%) (17, 21). Despite it established role as a cornerstone in MM management, ASCT remains underutilized in Colombia due to structural and socioeconomic barriers, with around 39.9% of eligible patients under 65 years undergoing transplant in prior studies (23). Patients face multiple barriers including informational, financial, logistical, and cultural (24).

Survival outcomes in this study were comparable to other real-world data, although differences in patient populations and treatment regimens should be considered. For instance, while the median OS in our cohort was 27.5 months, studies in the U.S. have shown OS between 10.9 and 15.4 months depending on regimen (22). Similarly, the median PFS in our cohort was 14.6 months, which is longer than that observed in some clinical trials in heavily pretreated populations, such as studies evaluating daratumumab in refractory MM that reported shorter PFS (3.7 months) (25). These differences highlight the variability between clinical trial populations and real-world settings, as well as the importance of real-world evidence to complement clinical trial findings.

Real-world studies have shown improved outcomes with newer agents (22, 26, 27) and underscore the importance of integrating these therapies into disease management, especially when novel drugs have been recently approved (5, 28). Despite approvals, access remains unequal across LATAM, often limited by costs, reimbursement policies, and clinical trial availability (4, 29). Availability of therapies does not guarantee real-world use, and local policies can significantly limit access. In addition, our findings support calls for clinical practice guidelines that reflect local realities and establish clear treatment pathways for relapsed MM (22, 30). Efforts to bridge the gap between clinical research and the applied use of new medications in routine practice to enhance the outcomes for patients diagnosed with MM.

Our findings also highlight variability in time to treatment initiation. Although the average time from diagnosis to first-line therapy was 21 days, the wide range observed indicates heterogeneous patient experiences. A U.S.-based study similarly found that treatment delays were associated with factors such as gender, age, and healthcare setting (31). These findings underscore the need not only to expand access to novel therapies but also to improve the timeliness of care. Ensuring prompt initiation of treatment in accordance with clinical practice guidelines (CPGs) should be a key priority in the management of patients with MM.

Regarding safety, the 11.9% TEAE rate was primarily driven by hematologic events such as anemia and neutropenia. However, given the retrospective nature of the study and reliance on medical record documentation, this frequency may reflect underreporting rather than the true incidence of AEs. Similar variability in reported adverse event rates has been described in other retrospective studies, including analyses from China (32), where AEs occurred in 19% of patients and included leukopenia, thrombocytopenia, and neutropenia (33). Such differences in reported frequencies may be influenced by variations in documentation practices rather than true differences in safety profiles.

Colombia’s healthcare system is characterized by a mixed model of public and private financing, where individuals are affiliated mainly through three regimens: the contributory regime, the subsidized regime, and the special regime. These affiliation regimens represent populations with different socioeconomic conditions. The contributory regime includes individuals with the ability to pay, such as employees and self-employed workers, whereas the subsidized regime covers low-income individuals (34). Previous research in the country has shown differences in the occurrence of diseases and the use of health services between these regimens (35). Most patients in this study belonged to Colombia’s contributory healthcare regimen. Given the country’s segmented system, this may limit generalizability to populations covered by the subsidized or special regimens.

Finally, no QoL data were available in medical records, precluding the evaluation of patient-reported outcomes and the broader impact of treatment beyond clinical endpoints. This likely reflects the lack of systematic QoL assessment in routine clinical practice, representing an important real-world evidence gap in this setting.

## Conclusion

In this real-world cohort of patients with MM who experienced a second relapse and received third-line therapy, treatment patterns evolved across lines of therapy. Bortezomib-based regimens were predominantly used in the frontline setting, lenalidomide-based combinations became more common in the second line, and the third line was characterized by increasing heterogeneity with greater use of regimens based on novel agents. A substantial proportion of patients underwent ASCT, and lenalidomide was the most frequently used maintenance therapy after ASCT.

Effectiveness outcomes in the third-line setting indicated that nearly half of the patients achieved an objective response. Median OS exceeded two years from the initiation of treatment for the second relapse, and median progression-free survival was longer than one year. Treatment-emergent adverse events were reported in a minority of patients and were mainly hematologic. Data on health-related QoL were not available due to the absence of systematic assessment in routine clinical practice, highlighting an important gap in the clinical information captured in this real-world setting. Overall, these results offer a detailed characterization of treatment patterns, effectiveness, and safety outcomes in patients receiving third-line therapy after a second relapse.

### Limitations

This study has several limitations. First, the retrospective design relies on data extracted from medical records, which may be incomplete or inconsistently documented, especially for variables such as adverse events and quality of life. Although efforts were made to standardize data collection, missing information limited further analysis. In particular, given the reliance on routine clinical documentation, there is a potential for underdocumentation and undercapture of adverse events, especially for mild events or those not systematically recorded in clinical practice. Therefore, the reported frequency of TAES should be interpreted with caution, as it may underestimate the true incidence.

Second, the study focused exclusively on patients in second relapse who had previously received both lenalidomide and proteasome inhibitors, which narrows generalizability but also represents a high-risk subgroup with limited evidence available. In our time to event analysis death in the absence of documented progression represents a relevant competing event. Kaplan–Meier estimators assume non-informative censoring and may therefore overestimate the cumulative incidence of the event of interest in the presence of competing risks. As a result, the reported estimates for PFS should be interpreted with caution.

Third, while patients were recruited from five major institutions across different cities in Colombia, the majority were affiliated with the contributory healthcare regime. Therefore, results may not fully reflect the experience of patients under the subsidized or special regimes, who may face additional barriers to treatment access.

Moreover, the lack of reported data on QoL limited our ability to explore this exploratory endpoint. Although QoL assessment was planned, the absence of this information in the clinical records prevented meaningful analysis. Given the chronic nature of MM and the impact of treatment on daily functioning, future prospective studies should include validated QoL instruments to better capture patient-reported outcomes.

An additional limitation is the relatively small sample size, which restricted the ability to perform multivariable regression analyses to adjust for potential confounding factors. Therefore, the findings are based on descriptive analyses and should be interpreted with caution, without drawing causal or comparative conclusions between treatment strategies.

Despite the limitations, the multicenter design and the use of real-world data provide valuable insights into current practices in Colombia. This study highlights the need for improved access to novel agents, better documentation practices, and the development of national guidelines adapted to regional contexts. It also emphasizes the importance of expanding real-world research to support decision-making and improve outcomes in relapsed MM.

## Data Availability

The original contributions presented in the study are included in the article/**Supplementary Material**. Further inquiries can be directed to the corresponding author.
