# Correction: Treatment patterns and outcomes in patients with multiple myeloma in second relapse in Colombia (freedomm): a multicenter observational study

**DOI:** 10.3389/fonc.2026.1938900

**Published:** 2026-07-28

**Authors:** Guillermo Quintero, Sandra Aruachan, Jair Figueroa Emiliani, Kenny Galvez, Luis Antonio Salazar, Constanza Otero Venegas, Diana Buitrago, Humberto Martínez-Cordero, Juliana Saavedra, Maria Fernanda Vargas

**Affiliations:** 1 Fundación Santa Fe de Bogotá, Bogotá, Colombia; 2 Instituto Médico de Alta Tecnología Oncomédica, Montería, Colombia; 3 Hospital Mayor Méderi, Bogotá, Colombia; 4 Hospital Pablo Tobón Uribe, Medellín, Colombia; 5 Hematology and Hematopoietic Stem Cell Transplantation Unit, Clinica FOSCAL, Floridablanca, Santander, Colombia; 6 Facultad de Ciencias de la Salud, Universidad Autónoma de Bucaramanga, Bucaramanga, Colombia; 7 Real-World Evidence – Epidemiology & Clinical Data Management, Latin America, Central America and the Caribbean, Bogotá, Colombia; 8 Centro de Excelencia en Mieloma Múltiple - Unidad de Hemato-Oncología, Instituto Nacional de Cancerología, Bogotá, Colombia; 9 Unidad de Hematología y Trasplante de Médula Ósea, Hospital Militar Central, Bogotá, Colombia; 10 Sanofi, Bogotá, Colombia

**Keywords:** multiple myeloma, treatment patterns, real-world evidence, effectiveness, safety

**Figures 1** and **2** and their respective figure captions were in the wrong. The content displayed as **Figure 1** including its caption should have appeared as **Figure 2**, and the content displayed as **Figure 2** including its caption should have appeared as **Figure 1**. The order has now been corrected.

The original version of this article has been updated.

