# Supplementary material for: Treatment patterns and outcomes in patients with multiple myeloma in second relapse in Colombia (freedomm): a multicenter observational study
Source: Front Oncol. 2026 Jul 1;16:1831845. doi: 10.3389/fonc.2026.1831845 (PMC13368524; doi:10.3389/fonc.2026.1831845)
Supplement: Supplementary file 1 [file Table1.docx]

**Supplementary File 1. Outcomes definitions**

| Endpoint | Definition |
| --- | --- |
| PFS | The time from the date of start of the treatment pattern for second relapse to the date of disease progression or death from any cause, whichever occurs earlier. |
| OS | The time from the date of start of the treatment pattern for second relapse to the date of death due to any cause. Participants were censored at the date when the participant was last known to be alive or the data cut-off date, whichever occurred earlier. |
| ORR | The percentage of patients who achieved either a stringent complete response (sCR), complete response (CR), very good partial response (VGPR), or partial response (PR) with the treatment pattern for second relapse. |
| TEAE and Serious TEAE | Percentage of TEAE was defined as any unfavourable medical event that the investigator classified as related to the use of a drug included in the treatment patterns for the second relapse (i.e. IMiDs, PIs, MAbs, HDACIs). |
|  | Percentage of Serious adverse event was defined as any unfavourable medical event that results in any of the following: Death, life-threatening, inpatient hospitalization, or prolongation of existing hospitalization, persistent or significant disability/incapacity, or a congenital anomaly/birth defect. |
| Treatment discontinuation due to TEAE | Percentage of subjects discontinued due to TEAE. |

**Supplementary Table 1. Distribution of Patients by Treatment Type and Description of Medications Administered**

| Parameters | N (%) |
| --- | --- |
|  |  |
| Patient receiving autologous stem cell transplantation, N (%) | |
| Yes | 31 (36.9%) |
| No | 53 (63.1%) |
| Patient receiving any medication, N (%) |  |
| Yes | 25 (29.8%) |
| No | 59 (70.2%) |
| Medications description, N (%)* | **N=33** |
| Bortezomib | 3 (9.1%) |
| Daratumumab | 1 (3.0%) |
| Dexamethasone | 5 (15.2%) |
| Lenalidomide | 15 (45.5%) |
| Thalidomide | 6 (18.2%) |
| Other - Zoledronic acid | 3 (9.1%) |
| ** Percentages are calculated using the total number of prescriptions (not patients) as denominator, as single patient might receive more than one medication*  *Certain treatments for multiple myeloma are not included in the table because they were not found in the analyzed cohort (such as Belantamab mafodotin-blmf, Bendamustine (Treanda), Carfilzomib, Cyclophosphamide, Cisplatin, Doxorubicin, Liposomal doxorubicin, Elotuzumab, Etoposide, Ixazomib, Melphalan, Melphalan flufenamide, Panobinostat, Pomalidomide, Prednisone, Selinexor, Vincristine)* | |

**Supplementary Table 2. Effectiveness of treatment patterns for second relapse, according to the time to second relapse.**

| Parameters | Time to second relapse | | | |
| --- | --- | --- | --- | --- |
|  | **<6 months** | **6-12 months** | **12-24 months** | **≥ 24 months** |
| Response to third-line therapy, N (%) | |  |  |  |
| ORR* | 7 (46.7%) | 5 (55.6%) | 9 (56.2%) | 3 (27.3%) |
| OS with third-line therapy** |  |  |  |  |
| Median (95% CI) | 15.8 (6.7 - NE) | 27.5 (11.4 - NE) | 40.4 (18.07 - NE) | NR (26.8 - NE) |
| PFS with third-line therapy*** |  |  |  |  |
| Median (95% CI) | 9.9 (6.7 - 22.0) | 11.4 (5.82 - NE) | 22.3 (13.7 - 51.2) | 14.6 (7.27 - NE) |
| TTF with third-line therapy**** |  |  |  |  |
| Median (95% CI) | 5.6 (2.7 - 20.9) | 5.8 (3.3 -14.4) | 5.9 (3.0 - 24.0) | 7.2 (2.4 - NE) |
| *SD: Standard Deviation, IQR: Interquartile Range; Min: Minimum; Max: Maximum. Percentages are calculated using non-missing values as denominator. * ORR= percentage of patients with either sCR, CR, VGPR or PR of patients with MM treated with the different treatment patterns for second relapse.** OS= time from the date of start of second-relapse treatment to the date of death from any cause. *** PFS= time from the date of start of third-line therapy to the date of progressive disease or death from any cause. **** TTF= time from the date of start of third-line therapy until treatment discontinuation due to disease progression, treatment intolerance, or other reasons.NR = Not reached,   NE = Not estimable, the upper confidence limit could not be calculated due to censoring.* | | | | |
